# Study on Acoustic Properties of Helmholtz-Type Honeycomb Sandwich Acoustic Metamaterials

**DOI:** 10.3390/ma18071600

**Published:** 2025-04-01

**Authors:** Xiao-Ling Gai, Xian-Hui Li, Xi-Wen Guan, Tuo Xing, Ze-Nong Cai, Wen-Cheng Hu

**Affiliations:** Institute of Urban Safety and Environmental Science, Beijing Academy of Science and Technology, Beijing 100054, China; lixianh@vip.sina.com (X.-H.L.); guan0037@foxmail.com (X.-W.G.); xingtuo1991@163.com (T.X.); who518@126.com (W.-C.H.)

**Keywords:** honeycomb structures, resonant structure, acoustic metamaterial, sound absorption, transmission loss

## Abstract

In order to improve the acoustic performance of honeycomb sandwich structures, a Helmholtz-type honeycomb sandwich acoustic metamaterial (HHSAM) was proposed. The theoretical and finite element models were established by calculating the acoustic impedance of multiple parallel Helmholtz resonators (HR). By comparing the sound absorption of the single and multiple HR, it was found that the simulation results were basically consistent with the theoretical calculations. The sound absorption and insulation performance of the honeycomb panels, the honeycomb perforated panels, and the HHSAM structure were compared through impedance tube experiments. The results showed that, over a wide frequency range, the acoustic performance of the HHSAM structure was superior to that of the other two structures. Under scattered sound field conditions, the reverberation room results showed that the sound absorption of the HHSAM structure was better than that of the honeycomb panel in the frequency range of 100–5000 Hz. The noise reduction coefficient (NRC) of the honeycomb panel was 0.1, indicating almost no sound absorption effect in engineering. The NRC of the HHSAM structure could reach 0.35. In terms of sound insulation, the HHSAM structure was more prominent in the 400–4000 Hz range than the honeycomb panel. In the frequency range of 500–1600 Hz, the transmission loss of the HHSAM was 5 dB higher than that of the honeycomb panel.

## 1. Introduction

Noise pollution mainly includes industrial noise [1], traffic noise [2], construction noise [3], and social noise [4]. With the rapid development of the economy and society, severe noise pollution has become a major concern. In industrial areas, transportation hubs, and densely populated urban areas, this noise pollution is particularly severe. Noise pollution not only disturbs the environment but also poses a major threat to public health. Noise can lead to mood disturbances, emotional instability, reduced tolerance, irritability, increased blood pressure, and long-term exposure to noise pollution, which can cause heart disease and tinnitus, thus reducing people’s life expectancy [5]. Noise pollution has become a significant environmental concern in many large cities globally [6,7,8]. Therefore, the question of how to effectively control noise has become an important issue in the fields of environmental protection and public health.

In order to cope with noise pollution, scientists and engineers have developed various noise control methods. There are basically two methods for noise control: passive control technology [9] and active control technology [10]. A combination of these two methods can also be used. Active noise control technology can effectively suppress low-frequency noise, but its cost is high and the system is complex. The passive noise control method mainly uses techniques such as sound absorption, sound insulation, and noise reduction on the propagation path of noise to prevent the propagation of sound. Traditional sound-absorbing materials, such as porous foam and fiber materials, perform well in the middle and high frequencies but have poor acoustic performance in the low frequencies. Sound insulation technology relies on the quality and thickness of materials. To control low-frequency noise, it is necessary to use heavy and thick materials. With the increasing demand for lightweight components in aerospace, the automotive industry, and green buildings, the development of acoustic structures that combine efficient noise reduction and lightweight characteristics has become a research focus.

The honeycomb sandwich structure is widely used in aerospace, railway vehicles, ships, automobiles, construction, and other fields [11,12], because of its excellent performance, including its light weight, high specific strength, high specific stiffness, good stability, thermal insulation, and so on. Considering that the working environment in which the structure is located is often accompanied by noise and vibration, excessive noise not only causes discomfort to relevant personnel but also affects the service life of structural equipment. However, limited by the mass law, the honeycomb sandwich structure has a major disadvantage [13]. In terms of acoustic performance, compared with traditional materials such as aluminum alloys, honeycomb sandwich structures have much poorer sound insulation performance than homogeneous panels of the same quality, mainly because the coincidence frequency of sandwich panels occurs in a wider frequency range [14].

Researchers have undertaken significant investigations to boost the acoustic performance of honeycomb sandwich structures, focusing on the following key strategies: improving the stiffness of honeycomb sandwich structures [15,16]; adding porous materials [17], damping materials [18], or gas layers [19,20] inside the honeycomb; creating micropores on honeycomb sandwich structures [21]; and introducing acoustic metamaterials [13,22,23,24,25]. In addition, the negative stiffness and nonlinear effects can also dissipate a part of the system energy of the sandwich structure to reduce vibration and noise [26,27]. Most of the above studies were conducted using small samples under vertical incidence and verified through impedance tube experiments. Peiffer et al. [23] suggested that the installation conditions of the sample in the impedance tube were not consistent with the actual installation conditions of the engineering size panel. In addition, in impedance tubes, it is a plane wave, while, in engineering, it is a scattered sound field. Thus, the transmission loss (TL) of the structure measured in the impedance tube under vertical incidence cannot be directly compared with the TL of the scattered sound field environment in the field.

In order to improve the acoustic performance of honeycomb sandwich structures and provide a reference for practical engineering applications, this paper proposes a Helmholtz-type honeycomb sandwich acoustic metamaterial (HHSAM) that replaces each honeycomb in a honeycomb sandwich structure with a small Helmholtz resonator (HR), utilizing the resonance of Helmholtz resonator units and their interactions to improve the acoustic performance of the honeycomb sandwich structure. The overall structure achieves good acoustic effects while being lightweight.

## 2. Structure of the HHSAM

**Figure 1:**
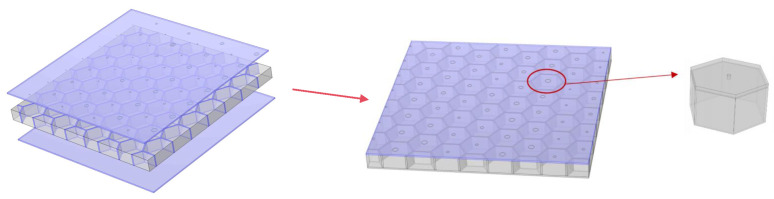
The Helmholtz-type honeycomb sandwich acoustic metamaterial.

The HHSAM structure consists of a top plate, honeycomb core, and bottom plate, as shown in Figure 1. The top plate is composed of a 1-mm-thick metal aluminum plate. We produce small holes with a periodic distribution on the top plate through mechanical punching or laser punching technology. The honeycomb core is composed of resin and created through 3D printing; alternatively, an aluminum honeycomb core can be selected. The distribution of the holes on the top plate corresponds to the position of the honeycomb core, and there is a hole on the top plate corresponding to the center of the honeycomb core. The holes on the top plate are equivalent to the neck of the Helmholtz resonator, and the cavity of the honeycomb core is equivalent to the Helmholtz resonant cavity. Each small hole and the cavity of the honeycomb core below it form a Helmholtz resonator. The bottom plate is also composed of a 1 mm aluminum plate. This structure not only has the lightweight feature of a honeycomb sandwich structure but also has the characteristics of an acoustic metamaterial.

## 3. Sound Absorption of Multiple Helmholtz Units

### 3.1. Sound Absorption Theory

The HHSAM structure can be seen as a parallel structure consisting of multiple Helmholtz resonators with different resonant frequencies. The sound absorption coefficient of the HHSAM structure can be obtained by calculating the acoustic impedance of the multiple Helmholtz resonators. The theoretical sound absorption coefficient α of the single Helmholtz resonator can be calculated by

(1) $$\alpha \;=\;1\;-{\;|R|}^{2}\;.$$

The pressure reflection coefficient *R* can be estimated by

(2) $$R\;=\;\frac{Z_{si}-1}{Z_{si}+1}\;.$$

$Z_{si}$ (i = 1, 2, 3, …… n) is the normalized surface impedance of the Helmholtz resonator and can be determined by

(3) $$Z_{si}\;=\;Z_{HRi}/\sigma \;,$$

where

(4) $$Z_{HRi}\;=\;r_{HRi}+j\chi_{HRi}\;,$$

(5) $$\sigma \;=\;\pi r_{i}^{2}/S\;,$$

(6) $$S\;=3\sqrt{3}/2{\left[a+\left(\sqrt{3}/3\right)t\right]}^{2}\;,$$

(7) $$r_{HRi}\;=k\delta_{\nu }l^{\prime }/r_{i}+k\left(\gamma -1\right)\delta_{t}l/2/r_{i}\;,$$

(8) $$\chi_{HRi}\;=kl^{\prime }\left(1+\delta_{\nu }/r_{i}\right)-\sigma cot\left(k\;D\right)\;,$$

(9) $$k\;=\omega /c_{0}\;,$$

(10) $$\delta_{\nu }=\sqrt{2\nu /\omega }\;,$$

(11) ω=2πf,

(12) $$\delta_{t}\approx 0.25\times 10^{-2}/\sqrt{f}\;,$$

(13) $$l^{\prime }=l+l_{c1}+l_{c2}\;,$$

(14) $$l_{c1}\;=0.82r_{i}\left[1-1.35\left(r_{i}/a\right)+0.31{\left(r_{i}/a\right)}^{3}\right]\;,$$

(15) $$l_{c2}\;=0.82r\left[1-0.235\left(r_{i}/a\right)-1.32{\left(r_{i}/a\right)}^{2}+1.54{\left(r_{i}/a\right)}^{3}-0.86{\left(r_{i}/a\right)}^{4}\right]\;.$$

The following definitions apply. 

σ: the surface porosity of the single Helmholtz resonator.

*S*: the single Helmholtz resonator’s surface area (unit in m^2^).

$r_{i}$ (i = 1, 2, 3, …… n): the neck radius of the Helmholtz resonator (unit in m).

*a*: the edge length of the honeycomb (unit in m).

*t*: the honeycomb core wall thickness (unit in m).

*k*: the wavenumber.

$\delta_{\nu }$: the viscous boundary layer thickness (unit in m).

ν: the kinematic viscosity of air ($\nu \approx 15\times 10^{-6}$ m^2^/s).

γ: the Poisson constant of air (γ=1.4).

$\delta_{t}$: the thermal boundary layer thickness (unit in m).

*f*: the frequency (unit in Hz).

$l^{\prime }$: the neck length, taking two end corrections into account (unit in m).

D: the depth of the single Helmholtz resonator (unit in m). 

**Figure 2:**
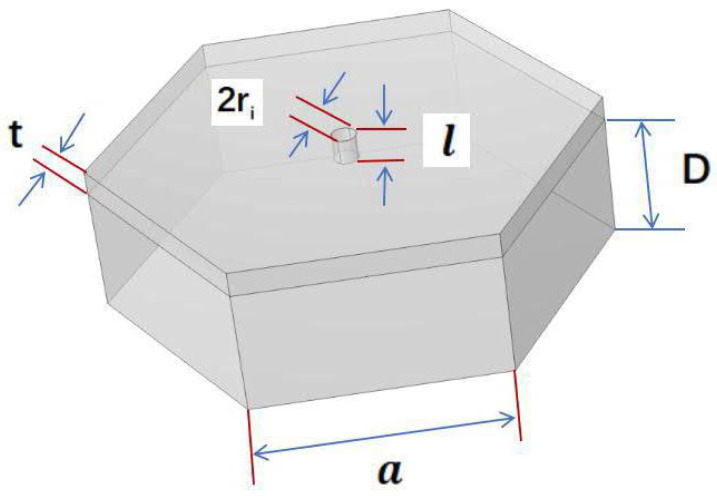
The structural parameters of the Helmholtz resonator cell.

The normalized surface impedance of the HHSAM structure can be derived as

(16) $$\frac{1}{Z_{ns}}\;=\;\sum_{i=1}^{n}\frac{1}{n(Z_{si})}\;,$$

and the absorption coefficient can be subsequently computed. The structural parameters of the Helmholtz resonator cell are shown in Figure 2.

### 3.2. Numerical Simulation

**Figure 3:**
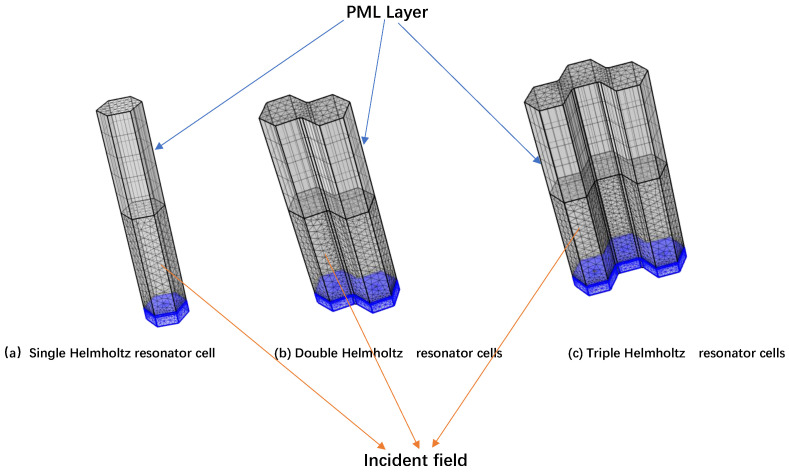
The finite element model of the single Helmholtz resonator cell, double Helmholtz resonator cells, and triple Helmholtz resonator cells.

To verify the theoretical models, finite element analysis is used to calculate the sound absorption of the Helmholtz resonator cell using the COMSOL Multiphysics 5.2 software. The 3D model is used for geometric modeling. The Helmholtz resonator cell is hexagonal with a side length of 10 mm. A small hole is opened on the top surface as the neck of the Helmholtz resonator cell. Figure 3 shows the finite element model of a single Helmholtz resonator cell, double Helmholtz resonator cells, and triple Helmholtz resonator cells. In the simulations, the thermoviscous acoustics modules are used to create a calculation program. The plane wave is applied to the incidence field. The far field is a perfect matching layer that can absorb all energy. The neck length of the Helmholtz resonator cell is 1 mm. The neck diameter of the single Helmholtz resonator cell is 0.5 mm (as shown in Figure 3a). The neck diameters of the double Helmholtz resonator cells are 0.5 mm and 0.7 mm (as shown in Figure 3b). The neck diameters of the triple Helmholtz resonator cells are 0.5 mm, 0.7 mm, and 1.0 mm (as shown in Figure 3c).

**Figure 4:**
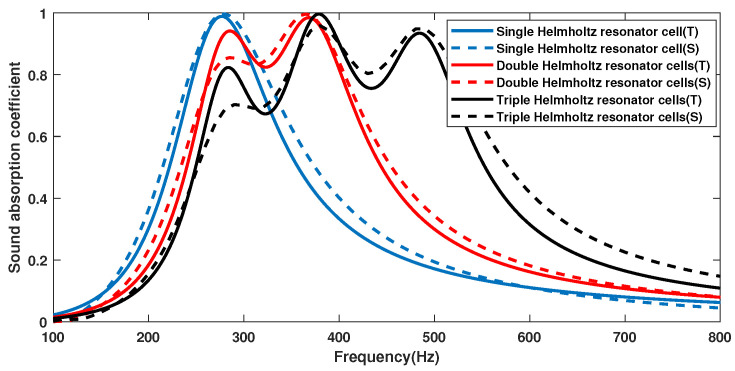
The sound absorption of a single Helmholtz resonator cell, double Helmholtz resonator cells, and triple Helmholtz resonator cells obtained through theoretical and numerical simulations.

**Figure 5:**
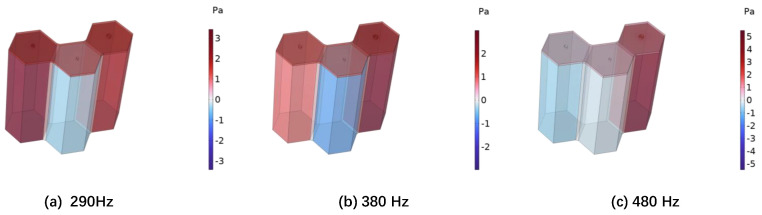
The sound pressure distribution of the triple Helmholtz resonator cells at three peak frequencies.

Figure 4 shows the sound absorption performance curves of the single Helmholtz resonator cell, double Helmholtz resonator cells, and triple Helmholtz resonator cells obtained through theoretical and numerical simulations. The solid line represents the theoretical calculation result. The dashed line is the result of the numerical simulation. It can be seen that, regardless of whether we consider a single Helmholtz resonator cell, double Helmholtz resonator cells, or triple Helmholtz resonator cells, the theoretical calculation and numerical simulation results are consistent. Both the theoretical and numerical simulation results show that using multiple Helmholtz resonator cells can significantly broaden the sound absorption bandwidth of the structure. For example, through theoretical analysis, it can be seen that the sound absorption coefficient of a single Helmholtz resonator cell exceeds 0.5 in the frequency range of 221 Hz–356 Hz, the range of double Helmholtz resonator cells is 240 Hz–445 Hz, and the range of three Helmholtz resonator cells is expanded to 250 Hz–553 Hz. Figure 5 shows the sound pressure distribution at the absorption peak for the triple Helmholtz resonator cells. It can be seen that the three Helmholtz resonator cells play different roles at different frequencies.

## 4. Experimental Study on the HHSAM Structure’s Acoustic Performance

### 4.1. Acoustic Performance Under Vertical Incidence

#### 4.1.1. Sound Absorption

**Figure 6:**
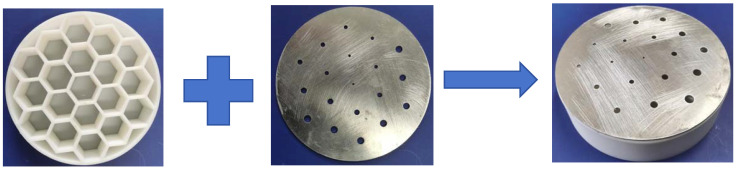
The sample of the HHSAM structure.

**Figure 7:**
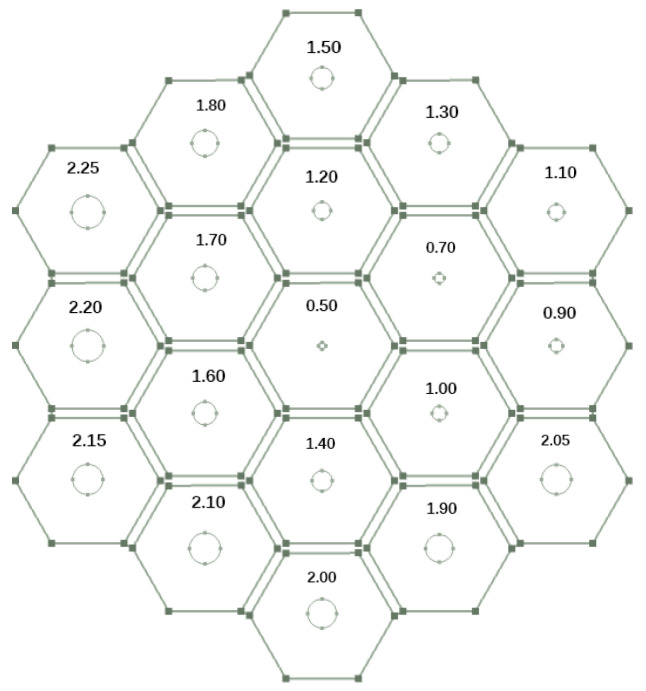
The neck size and distribution of the sample of the HHSAM structure.

**Figure 8:**
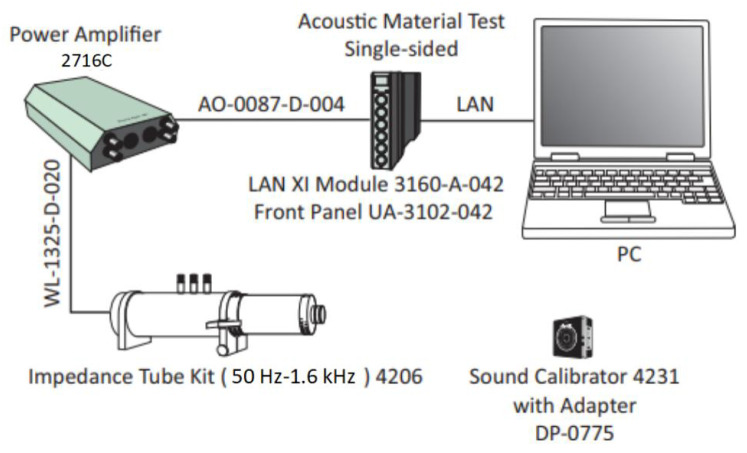
The test system of the impedance tube.

**Figure 9:**
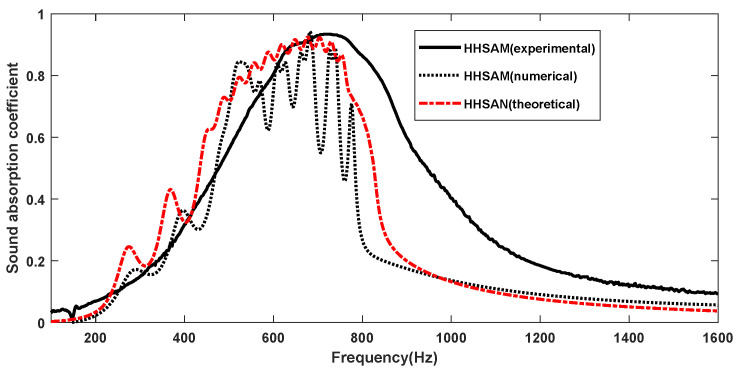
The theoretical, numerical, and experimental sound absorption curves of the HHSAM structure.

**Figure 10:**
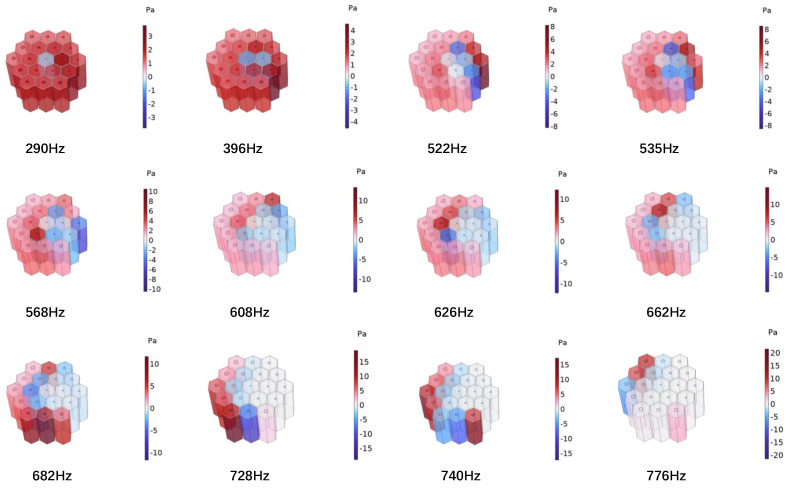
The sound pressure distribution of the HHSAM structure at the peak frequency.

Based on the theoretical and simulation results given in the previous section, it was found that parallel multiple Helmholtz resonators could achieve broadband sound absorption within the structure. Therefore, a HHSAM structure consisting of 19 Helmholtz resonators was designed. The HHSAM structure was constructed using parallel Helmholtz structures with different resonance frequencies, and we arranged them according to a certain periodic rule. The bottom and core layers of the HHSAM structure were created using 3D printing technology. The top panel was created by machining small holes of different diameters on a 1-mm-thick aluminum panel. Then, the two were combined through latex stations to form a HHSAM structure. The sample of the HHSAM structure is shown in Figure 6. The diameter of the sample is 100 mm. The height of the sample is 50 mm. The side length of a hexagonal honeycomb is 10 mm. The wall thickness of the honeycomb core is 2 mm. There is a hole on the top panel corresponding to each honeycomb, which represents the neck of the Helmholtz resonator. The size and distribution of the neck are shown in Figure 7 (unit in mm). A two-microphone impedance tube (type 4206) from Bruel Kjaer is applied to measure the normal incident absorption coefficient according to the standard procedure detailed in ISO (10534-2). The frequency range of measurement is from 100 to 1600 Hz. Figure 8 shows the test system of the impedance tube. Figure 9 shows the sound absorption performance of the HHSAM structure obtained through theoretical and numerical simulations and impedance tube experiments. It can be seen that the scope of action of the theoretical calculations and numerical simulations is basically consistent. The experimental results deviate slightly from the theoretical and numerical simulation results, which may be caused by manufacturing errors in the samples. Figure 10 shows the sound pressure distribution of the HHSAM structure at the peak absorption frequency. It can be seen from the distribution of the sound pressure that different Helmholtz resonator cells play different roles at different frequencies. Moreover, each Helmholtz resonance cell has a more or less mutual influence on other nearby cells. In summary, the theoretical, numerical, and experimental results effectively demonstrate that the HHSAM structure has good sound absorption performance over a wide frequency band range.

**Figure 11:**
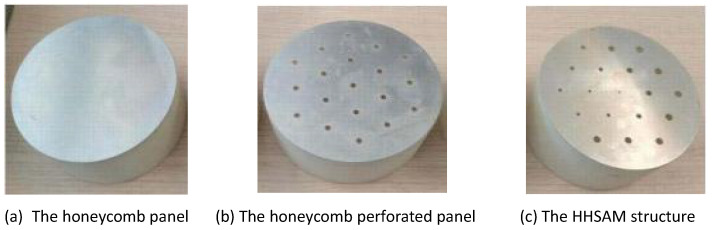
The samples of the honeycomb panel, the honeycomb perforated panel, and the HHSAM structure.

**Figure 12:**
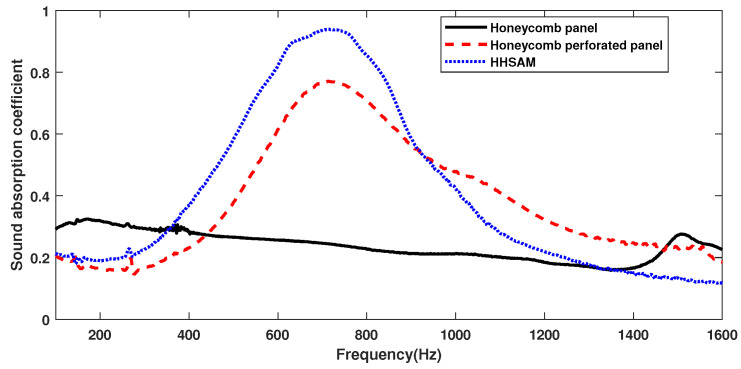
The experimental absorption of the honeycomb panel, the honeycomb perforated panel, and the HHSAM structure.

**Figure 13:**
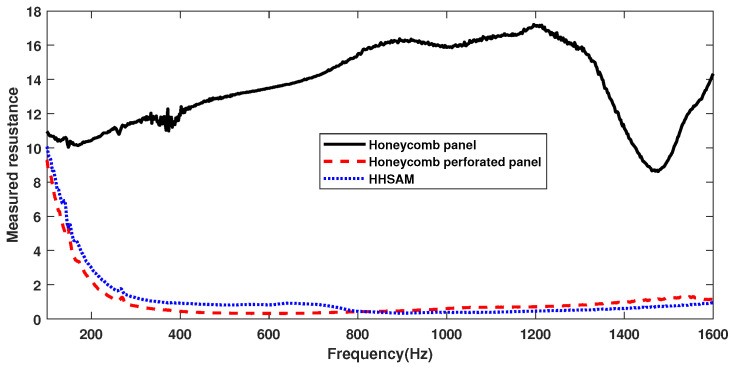
The measured resistance of the honeycomb panel, the honeycomb perforated panel, and the HHSAM structure.

**Figure 14:**
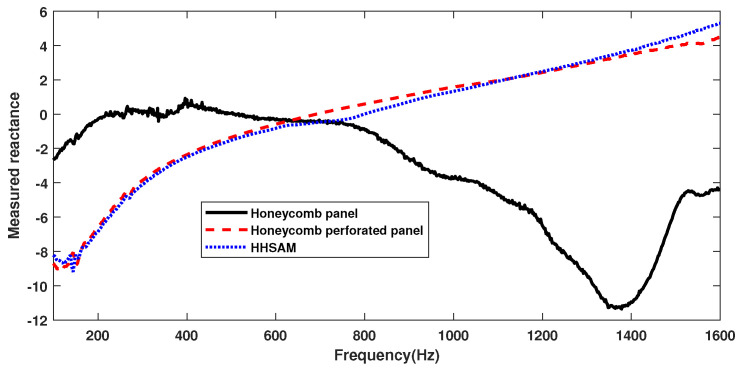
Themeasured reactance of the honeycomb panel, the honeycomb perforated panel, and the HHSAM structure.

For comparison, the acoustic performance of the honeycomb panels, the honeycomb perforated panels, and the HHSAM structure is tested separately. The thickness of all three structures is 50 mm, with only the top plate being different, as shown in Figure 11. Figure 11a shows a honeycomb panel structure, with a top plate composed of 1-mm-thick aluminum. Figure 11b shows the honeycomb perforated panel structure, with a top plate of a 1-mm-thick perforated panel and a perforated radius of 1.5 mm. Figure 11c shows the HHSAM structure, with a top plate thickness of 1 mm and 19 holes of different diameters distributed on top. Figure 12 shows the normal-incidence sound absorption coefficients of the honeycomb panel, the honeycomb perforated panel, and the HHSAM structure. It can be seen that the sound absorption performance of the honeycomb perforated panel and the HHSAM structure is significantly better than that of the honeycomb panel structure in the frequency range of 400 Hz–1600 Hz. The honeycomb perforated panel structure, with uniform holes on the upper panel, has a maximum sound absorption coefficient of 0.77 at 720 Hz, and the sound absorption coefficient is greater than 0.5 in the frequency range of 552 Hz–946 Hz. The HHSAM structure, with non-uniform holes on the upper panel, has a maximum sound absorption coefficient of 0.94 at 716 Hz, and the sound absorption coefficient is greater than 0.5 in the frequency range of 464–946 Hz. Thus, the HHSAM structure exhibits higher absorption coefficients and a wider absorption bandwidth. Figure 13 and Figure 14 depict the measurement results regarding the normalized surface impedance of the honeycomb panel, the honeycomb perforated panel, and the HHSAM structure. The experimental results indicate that the normalized surface resistance of the honeycomb panel is too high. The surface acoustic resistance of the honeycomb perforated panel and the HHSAM structure varies around 1 in the range of 400 Hz–1600 Hz. The surface acoustic reactances of the honeycomb perforated panel and the HHSAM structure are close to 0 at the peak of sound absorption.

#### 4.1.2. Sound Insulation Property

**Figure 15:**
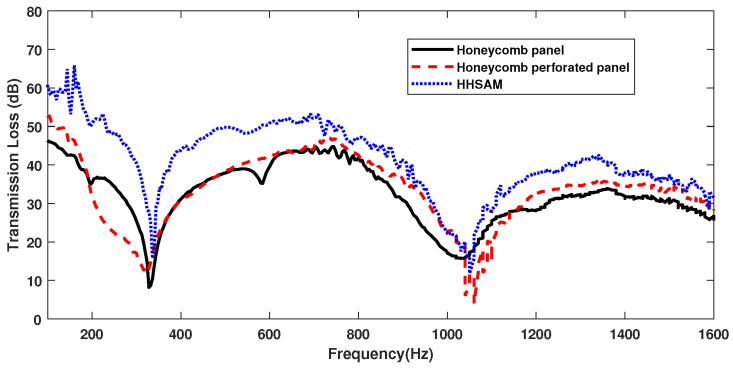
The TL of the honeycomb panel, the honeycomb perforated panel, and the HHSAM structure.

Figure 15 shows the sound insulation performance of the honeycomb panel, the honeycomb perforated panel, and the HHSAM structure under vertical incidence by an impedance tube. It can be seen that, besides the narrow frequency range around 1050 Hz, the TL of the HHSAM structure in the frequency range of 100 Hz–1600 Hz is about 10 dB higher than that of the honeycomb panel structure. The TL values of the honeycomb and honeycomb perforated panels are generally similar in the range of 352 Hz–512 Hz. The TL of the honeycomb perforated panel in the ranges of 690 Hz–1040 Hz and 1160 Hz–1600 Hz is about 3 dB higher than that of the honeycomb panel structure. Overall, the TL of the HHSAM structure is higher than that of the honeycomb and honeycomb perforated panels.

### 4.2. Acoustic Performance Under Scattered Sound Field Conditions

#### 4.2.1. Sound Absorption

The reverberation chamber method is carried out to measure the acoustic absorption coefficients of the honeycomb panel and the HHSAM structure under scattered sound field conditions, which are close to the reality in engineering practice. The samples are measured in a 226 m^3^ reverberation chamber based on ISO354. There are spherical crown diffusers at two interfaces in the reverberation chamber. The interior wall, floor, and ceiling of the reverberation chamber are all covered by smooth and hard ceramic tiles, which have strong emission effects and can be considered as having a sound absorption coefficient of 0. The equivalent sound absorption area of the test specimen $A_{T}$ can be calculated using the formula

(17) $$A_{T}\;=\;A_{2}\;-\;A_{1}\;=\;55.3\;V\;\left(\frac{1}{c_{2}\;T_{2}}-\frac{1}{c_{1}\;T_{1}}\right)-4\;V\;\left(m_{2}-m_{1}\right)$$

where $A_{1}$ is the equivalent sound absorption area of the empty reverberation room,

(18) $$A_{1}\;=\;\frac{55.3\;V}{c\;T_{1}}-4\;V\;m_{1}$$

*V* is the volume of the empty reverberation room, *c* is the propagation speed of sound in air, $T_{1}$ is the reverberation time of the empty reverberation room, and $m_{1}$ is the power attenuation, calculated according to ISO9613-1 using the climatic conditions that are present in the empty reverberation room during the measurement. The value of *m* can be calculated from the attenuation coefficient, α, which is used in ISO9613-1 according to the formula

(19) $$m_{1,2}\;=\;\frac{\alpha_{1,2}}{10lg(e)}$$

$A_{2}$ is the equivalent sound absorption area of the reverberation room containing a test specimen, which can be calculated using the formula

(20) $$A_{2}\;=\;\frac{55.3\;V}{c\;T_{2}}-4\;V\;m_{2}$$

$T_{2}$ is the reverberation time of the reverberation room after the test specimen has been introduced.

The sound absorption coefficient of a plane absorber of test objects can be calculated using the formula

(21) $$\alpha_{s}\;=\;\frac{A_{T}}{S}$$

where S is the area covered by the test specimen.

**Figure 16:**
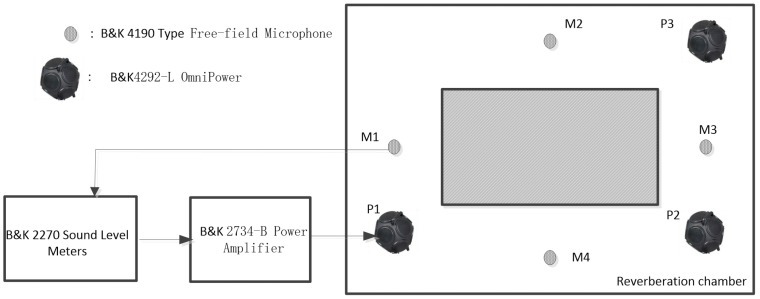
The schematic diagram of the reverberation chamber test.

**Figure 17:**
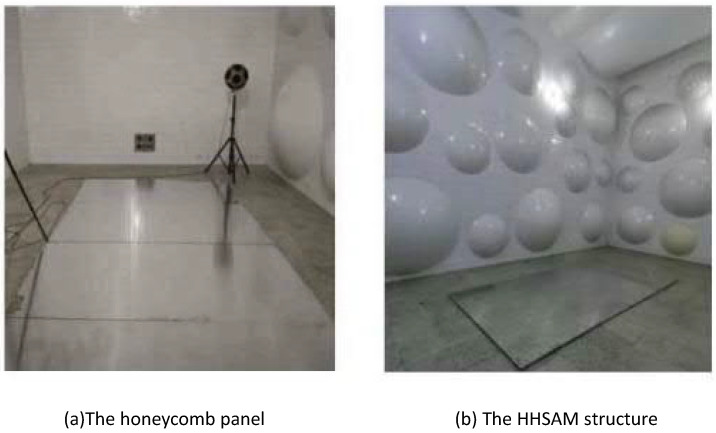
The reverberation chamber experiment using the honeycomb panel and the HHSAM structure.

**Figure 18:**
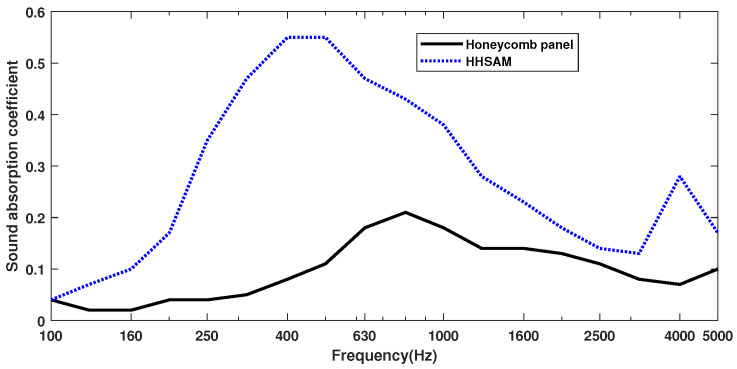
The sound absorption of the honeycomb panel and the HHSAM structure under scattered sound field conditions.

The sound absorption of the honeycomb panel and the HHSAM structure is measured for 1/3 octave frequency bands from 100 Hz to 5000 Hz in the reverberation chamber. The testing instrument adopts the 2270 analyzer system, a 4190 microphone, a 2734-B power amplifier, and a 4292-L dodecahedron sound source from Denmark’s B&K company (Naerum, Denmark). Figure 16 shows a schematic diagram of the reverberation chamber method used for testing. The sample of the HHSAM structure in the reverberation chamber is created by laser drilling on the upper plate of the aluminum honeycomb panel to create holes with a periodic distribution. The thickness of the honeycomb panel is 50 mm, and the thickness of the upper and lower cover panels is 1 mm. The aperture distribution is within the range of 0.5 mm–2.25 mm. The samples are placed in the middle of the reverberation chamber. Figure 17 shows the reverberation chamber experiment on the honeycomb panel and the HHSAM structure. Figure 17a shows the honeycomb panel structure, and (b) shows the HHSAM structure. The reverberation chamber’s temperature is 28 °C, and the relative humidity is 55%. Figure 18 shows the sound absorption performance of the honeycomb panel and the HHSAM structure under scattered sound field conditions. The maximum sound absorption coefficient of the honeycomb panel is close to 0.2, with a noise reduction coefficient NRC=0.1, indicating that it is almost impossible to use as a sound-absorbing material in engineering applications. The HHSAM structure has significantly improved sound absorption performance compared to the honeycomb panel structure. The maximum sound absorption coefficient reaches 0.55, with a noise reduction coefficient NRC=0.35.

#### 4.2.2. Sound Insulation Property

**Figure 19:**
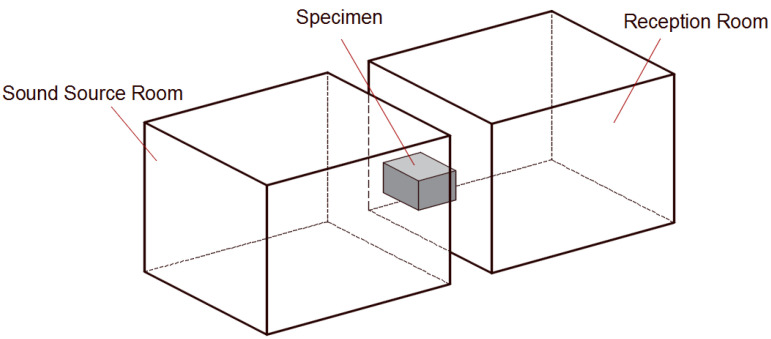
Schematic diagram of sound isolation measurement in the laboratory.

**Figure 20:**
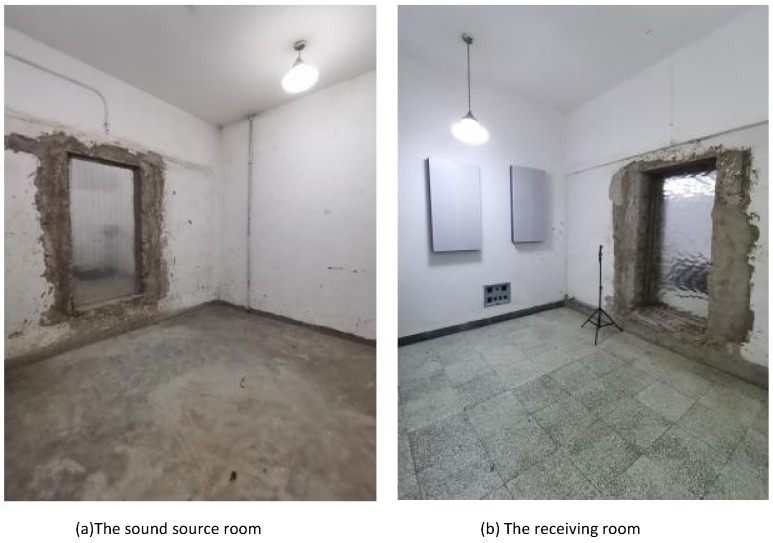
The sound source and receiving room.

**Figure 21:**
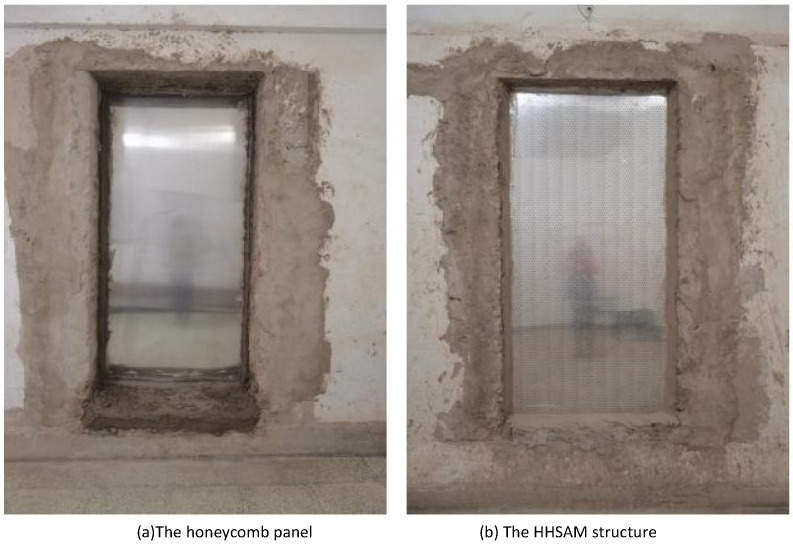
Sample installation for the honeycomb panel and HHSAM structure.

**Figure 22:**
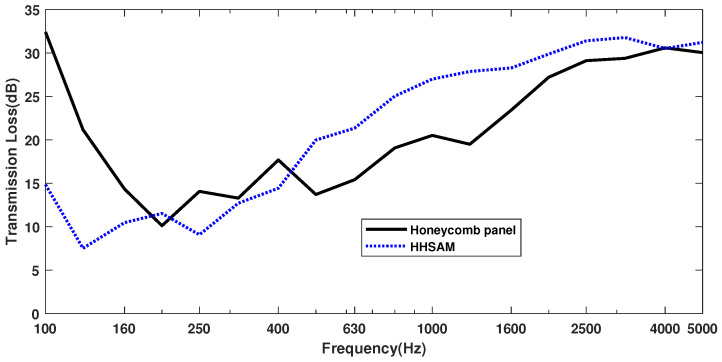
The TL of the honeycomb panel structure and the HHSAM structure.

The sound insulation quantity of the honeycomb panel and the HHSAM structure is measured in a sound insulation laboratory. The test facility consists of two adjacent chambers with a test opening between them, in which the test specimen is inserted. The volume of the sound source chamber and reception room is 83 m^3^ and 55 m^3^, respectively. The area of the specimen is 2.24 m^2^. The measurement is carried out in accordance with the test standards ISO 10140:2010 and ASTM E 90:2004. Two speakers are used to generate random noise in a source chamber. A half-inch microphone is used to measure the sound pressure levels at six microphone positions in each chamber. Figure 19 shows the schematic diagram of the sound isolation measurement in the laboratory. Figure 20 shows the actual sound source room (a) and the receiving room (b). Figure 21 shows the installation of the sample during the experimental process. Figure 21a is the honeycomb structure and (b) is the HHSAM structure. The insulation chamber’s temperature is 26 °C, and the relative humidity is 61%. Figure 22 shows the TL characteristic of the honeycomb panel and the HHSAM structure. Compared to the honeycomb panel structure, the HHSAM structure exhibits a higher TL value in the frequency range of 400 Hz–4000 Hz. At 1250 Hz, the TL of the HHSAM structure is 8 dB higher than that of the honeycomb panel.

## 5. Discussion

The research results show that honeycomb perforated panels have significantly improved acoustic performance compared to honeycomb panels. This conclusion is consistent with reference [21]. In contrast, this study further confirms this conclusion through impedance tube experiments (as shown in Figure 12). Under vertical incidence conditions, the maximum sound absorption coefficient of the honeycomb perforated panels can reach 0.77, which is much greater than the value of 0.3 for the honeycomb panel (Figure 12).

**Figure 23:**
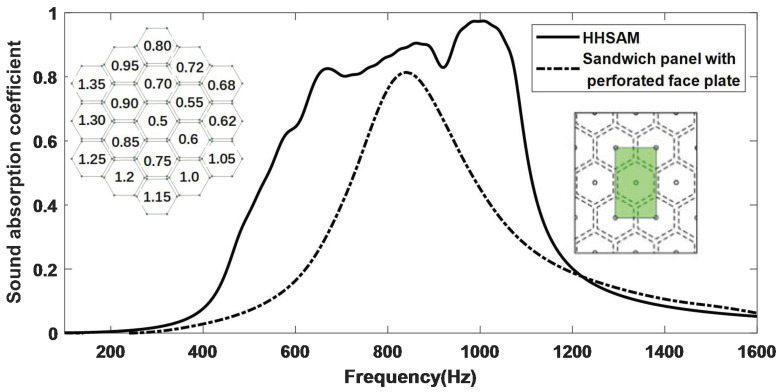
The acoustic performance of a 17-mm-thick sandwich panel with perforated faceplates and the HHSAM structure.

The perforation diameter of each structure in reference [21] is consistent, and their research mainly focuses on the influence of different types of honeycomb cores (hexagonal, rectangular, triangular) and the impact of changes in perforation on the acoustic performance of the structure. In contrast, in this study, we used different apertures with periodic distributions, theoretically forming more absorption peaks through different Helmholtz resonances, which could further expand the absorption bandwidth of the structure. We also verified this through experiments (as shown in Figure 12). The maximum sound absorption coefficient of the HHSAM structure can reach 0.94, which is higher than the 0.77 of the honeycomb perforated panel. The absorption bandwidth of the HHSAM structure with a sound absorption coefficient greater than 0.5 is also extended towards lower frequencies by about 100 Hz compared to the honeycomb panel structure. Compared with the 17-mm-thick hexagonal honeycomb core sandwich panel with perforated faceplates used in reference [21], we also theoretically calculated the sound absorption of a 17-mm-thick HHSAM structure, as shown in Figure 23. The perforation diameter in reference [21] was 1mm, while the perforation diameter of the HHSAM structure was between 1.0 mm and 2.7 mm. It can be seen that, under the same thickness, the sandwich panel with perforated faceplates reaches a maximum sound absorption coefficient of 0.82 at 840 Hz, and the sound absorption coefficient is greater than 0.5 in the range of 730 Hz–980 Hz. The maximum sound absorption coefficient of the HHSAM structure can reach 0.97 at 1002 Hz, and the sound absorption coefficient is greater than 0.5 in the range of 540 Hz–1109 Hz. This shows that using structures with different apertures with a periodic distribution can better improve the acoustic performance of honeycomb panel structures than using a single-aperture structure.

Unlike the existing literature, in order to facilitate engineering applications, this study produced a large sample of 2.24 m^2^ and conducted experiments with irregular incidence. The results also showed that the HHSAM structure had better acoustic performance than honeycomb panels (Figure 18 and Figure 22). In terms of the production process, only the top plate of the original honeycomb panel is replaced with a perforated panel with periodic distribution holes. This indicates that mass production is achievable.

Although these results are promising, there are still some challenges. To improve the acoustic performance of the HHSAM structure, it is necessary to further optimize the structural parameters based on the operating frequency. The impact of oblique incidence on the acoustic performance of HHSAM structures needs further analysis, and the influence of opening holes on the strength of honeycomb panels on the top surface also needs experimental evaluation.

## 6. Conclusions

In this study, the acoustic properties of the HHSAM structure were investigated in terms of theory, simulations, and experiments. The theoretical calculations were validated by comparing them with numerical results, and excellent agreement was achieved. The theoretical, simulation, and experimental results all demonstrate that a wide frequency range of sound absorption can be achieved by the parallel connection of multiple Helmholtz resonators with different resonant frequencies. The impedance tube experiments showed that the maximum sound absorption coefficient of the HHSAM structure could reach 0.9, which was much greater than the 0.3 of the honeycomb panel. At the same time, in the frequency range of 100 Hz–1600 Hz, the TL of the the HHSAM structure was 10 dB higher than that of the honeycomb panel structure. The honeycomb perforated panels also could not achieve this effect. Under scattered sound field conditions, the sound absorption and insulation performance of the HHSAM structure was also better than that of the honeycomb panel structure over a wide frequency band range. In the frequency range of 428 Hz to 3906 Hz, the TL of the HHSAM structure was significantly higher than that of honeycomb panels. At 1250 Hz, the TL of the HHSAM structure was 8 dB higher than that of honeycomb panels.

## Data Availability

The original contributions presented in this study are included in the article. Further inquiries can be directed to the corresponding author.

## References

[1] Wang Y.B., Xie J.L., Lee H.M., Lee H.P. (2025). Industrial noise reduction measures based on the Italian prioritisation index. J. Build. Eng..

[2] Danilevicius A., Karpenko M., Krivanek V. (2023). Research on the noise pollution from different vehicle categories in the urban area. Transport.

[3] Wu Z.F., Zhao X.Q. (2025). Reducing construction noise: Sound masking effect on soundscape dominated by construction noise. Int. J. Environ. Sci. Technol..

[4] Yang D.L., Liu X.H., Ren Z.C., Li M.N. (2022). Relation between noise pollution and life satisfaction based on the 2019 China social survey. Int. J. Environ. Res. Public Health.

[5] Kumar S., Xiang T.B., Lee H.P. (2020). Ventilated acoustic metamaterial window panels for simultaneous noise shielding and air circulation. Appl. Acoust..

[6] Quader M.A., Rahman M.M., Chisty M.A., Hattawi K.S.A., Islam E.A.M.K. (2024). Evaluation of noise pollution impact on health in Dhaka city, Bangladesh. Front. Public Health.

[7] Monazzam M., Karimi E., Abbaspour M., Nassiri P., Taghavi L. (2015). Spatial traffic noise pollution assessment—A case study. Int. J. Occup. Med. Environ. Health.

[8] Stanovsk M., Tomskov H., Slachtov H., Potuznkov D., Argalsov L. (2024). Health impact of environmental and industrial noise—A narrative review. Med. Pr..

[9] Stosiak M., Yatskiv I., Prentkovskis O., Karpenko M. (2025). Reduction of pressure pulsations over a wide frequency range in hydrostatic system. Machines.

[10] Lee H.M., Hua Y.T., Wang Z.M., Lim K.M., Lee H.P. (2021). A review of the application of active noise control technologies on windoes: Challenges and limitations. Appl. Acoust..

[11] He M.F., Hu W.B. (2008). A study on composite honeycomb sandwich panel structure. Mater. Des..

[12] Zheng X., Lee H., Weisgraber T.H., Shusteff M., DeOtte J., Duoss E.B. (2014). Ultralight, ultrastiff mechanical metamaterials. Science.

[13] Li Y.L., Zhang Y.L., Xie S. (2020). A lightweight multilayer honeycomb membrane-type acoustic metamaterial. Appl. Acoust..

[14] Huang W.C., Ng C.F. (1998). Sound insulation improvement using honeycomb sandwich panels. Appl. Acoust..

[15] Ng C.F., Hui C.K. (2008). Low frequency sound insulation using stiffness control with honeycomb panels. Appl. Acoust..

[16] Kang L.D., Liu B.L., An F.Y., Wei D.P. (2022). Mechanical and vibro-acoustic performance of sandwich panel with perforated honeycomb cores. J. Acoust. Soc. Am..

[17] Yang Y., Li B.B., Chen Z.F., Sui N., Chen Z., Saeed M.U., Li Y.F., Fu R., Wu C., Jing Y. (2016). Acoustic properties of glass fiber assembly-filled honeycomb sandwich panels. Compos. Part B.

[18] Boztoprak Y., Ünal M., Özada Ç., Kuzu E., Özer H., Ergin F., Yazıcı M. (2023). Sound insulation performance of honeycomb core aluminum sandwich panels with flexible epoxy-based foam infill. Compos. Struct..

[19] Rajaram S., Nutt S. (2006). Measurement of sound transmission losses of honeycomb partitions with added gas layers. Noise Control Eng. J..

[20] Naify C.J., Huang C.Z., Sneddon M., Nutt S. (2011). Transmission loss of honeycomb sandwich structures with attached gas layers. Appl. Acoust..

[21] Meng H., Galland M.A., Ichchou M., Xin F.X., Lu T.J. (2019). On the low frequency acoustic properties of novel multifunctional honeycomb sandwich panels with micro-perforated faceplates. Appl. Acoust..

[22] Peng X.Y., Ji J., Jing Y. (2018). Composite honeycomb metasurface panel for broadband sound absorption. J. Acoust. Soc. Am..

[23] Sui N., Yan X., Huang T.Y., Xu J., Yuan F.G., Jing Y.A. (2015). Lightweight yet sound-proof honeycomb acoustic metamaterial. Appl. Phys. Lett..

[24] Lu K., Wu J.H., Guan D., Gao N.S., Jing L. (2016). A lightweight low-frequency sound insulation membrane-type acoustic metamaterial. AIP Adv..

[25] Peiffer A., Grunewald M., Lempereur P., Ni S., Xiang Y., Huang T.Y., Xu J., Yuan F.G. (2015). Comment on “A lightweight yet sound-proof honeycomb acoustic metamaterial”. Appl. Phys. Lett..

[26] Meng H., Huang X.C., Chen Y.Y., Theodossiades S., Chronopoulos D. (2021). Structural vibration absorption in multilayered sandwich structures using negative stiffness nonlinear oscillators. Appl. Acoust..

[27] Yasser D., Mohamed A.E. (2024). Numerical and experimental investigation of negative stiffness beams and honeycomb structures. Eng. Struct..

